# Analysis of the Response Signals of an Electronic Nose Sensor for Differentiation between *Fusarium* Species

**DOI:** 10.3390/s23187907

**Published:** 2023-09-15

**Authors:** Piotr Borowik, Valentyna Dyshko, Rafał Tarakowski, Miłosz Tkaczyk, Adam Okorski, Tomasz Oszako

**Affiliations:** 1 Faculty of Physics, Warsaw University of Technology, ul. Koszykowa 75, 00-662 Warszawa, Poland; rafal.tarakowski@pw.edu.pl; 2 Ukrainian Research Institute of Forestry and Forest Melioration Named after G. M. Vysotsky, 61024 Kharkiv, Ukraine; valya_dishko@ukr.net; 3 Forest Protection Department, Forest Research Institute, ul. Braci Leśnej 3, 05-090 Sękocin Stary, Polandt.oszako@ibles.waw.pl (T.O.); 4 Department of Entomology, Phytopathology and Molecular Diagnostics, Faculty of Agriculture and Forestry, University of Warmia and Mazury in Olsztyn, Pl. Łódzki 5, 10-727 Olsztyn, Poland; adam.okorski@uwm.edu.pl

**Keywords:** gas sensor, application of e-nose, *Fusarium*, pathogen detection, odor differentiation

## Abstract

*Fusarium* is a genus of fungi found throughout the world. It includes many pathogenic species that produce toxins of agricultural importance. These fungi are also found in buildings and the toxins they spread can be harmful to humans. Distinguishing *Fusarium* species can be important for selecting effective preventive measures against their spread. A low-cost electronic nose applying six commercially available TGS-series gas sensors from Figaro Inc. was used in our research. Different modes of operation of the electronic nose were applied and compared, namely, gas adsorption and desorption, as well as modulation of the sensor’s heating voltage. Classification models using the random forest technique were applied to differentiate between measured sample categories of four species: *F. avenaceum, F. culmorum, F. greaminarum, and F. oxysporum*. In our research, it was found that the mode of operation with modulation of the heating voltage had the advantage of collecting data from which features can be extracted, leading to the training of machine learning classification models with better performance compared to cases where the sensor’s response to the change in composition of the measured gas was exploited. The optimization of the data collection time was investigated and led to the conclusion that the response of the sensor at the beginning of the heating voltage modulation provides the most useful information. For sensor operation in the mode of gas desorption/absorption (i.e., modulation of the gas composition), the optimal time of data collection was found to be longer.

## 1. Introduction

Fungi, including those of the genus Fusarium, produce a variety of volatiles, some of which are species-specific. This affords the possibility of recognizing them by smell. The ability to detect pathogens early offers greater potential for their control.

The concept of an electronic nose [1] consists of the application of an array of gas sensors with an overlapping scope of gas detection and pattern recognition methods. By using electronic nose, the recorded sensor’s response to the presence of measured odors does not lead to analyses of the chemical composition of analyzed samples and the identification of chemical components. The application of machine learning methods is used for the classification of samples or estimation of the odor intensity.

Different types of gas sensors, based on different physical phenomena, can be used to construct electronic noses. There are reports of devices using electrochemical [2], gravimetric [3], and optical [4] sensors. However, the cost of the device is an important consideration; the simple, low-cost devices are usually based on commercially available metal oxide (MOX) sensors [5].

Devices applying MOX sensors can basically operate in two modes. The most common mode is to record patterns of the sensor’s response to changes in the composition of the gas being measured. It should be noted that most commercially available sensors are designed for such an operating mode [6]. The other approach is to examine the response patterns of the sensors to the change in their operating heater supply voltage, which corresponds to a change in the temperature of the sensing element.

For the first mode of the sensor’s operation, special care is needed [7] in the design of a pneumatic system required to provide a change in the sensor’s exposure to different gases, ensuring the repeatability of measurement conditions and the rapidity of gas switching.

In the case of electronic noses operating in the mode of modulating the heating voltage of the MOX sensor, the shape and duration of the modulation is important. One of the most commonly used shapes is the rectangle [8,9,10,11]. The staricase-like profile has been used in other studies [12,13,14]. Triangular shapes [10,15,16] and sinusoidal modulation patterns [17] have also been reported. In other research [18], using a modulation profile with periodically changing amplitude and frequency was proposed. Meng et al. [19] reported the optimization of the rectangular shape of the modulation frequency of the heating temperature for the detection of drug-producing chemicals.

An important issue with such a wide range of proposals is that there is no clear guidance on the choice of the optimal profile of gas composition or the sensor’s heater voltage modulation. To the best of our knowledge, we are also not aware of direct comparisons of the performance of the electronic noses working in these two different modes of operation. One of the goals of the presented paper was to compare, using the same device and the same measured samples, an electronic nose applying both modes of operation. Another goal, from the perspective of the device’s construction, was to select the time of data collection required to differentiate the studied samples.

*Fusarium* is a genus of fungi found throughout the world. It includes many plant pathogens that produce toxins of agricultural importance. *Fusarium* include wilt, blight, rot, and canker of many garden, field, ornamental, and forest plants in agricultural and natural ecosystems. *Fusarium* also produces a variety of toxic secondary metabolites such as trichothecenes and fumonisins that can contaminate agricultural products, making them unsuitable for food or feed.

Fungi of the genus *Fusarium* are considered ubiquitous, i.e., they easily adapt to changing environmental conditions and develop over a wide temperature range, and this ability allows them to compete successfully with other species for ecological niches. For this reason, species of the genus *Fusarium* are very common in buildings. The source of spores in indoor air is mycelium growing on organic substrates and indoor surfaces. Their occurrence has been reported in building and finishing materials, i.e., in wood and paper products, mineral building materials, materials and paint coatings, surfaces of building partitions and elements of ventilation systems, and air filters, as well as in humidifiers and spray chambers. Direct exposure to the fungus or substances that it produces may lead to a deterioration in human health.

Research to limit the spread of fungi of the genus *Fusarium* has been conducted worldwide for many years [20]. One of the most commonly used methods to limit fungal growth is the use of bacteria of the genus *Bacillus* sp. [21,22]. However, not all species of the genus *Fusarium* react in the same way to the presence of bacteria.

**Figure A1:**
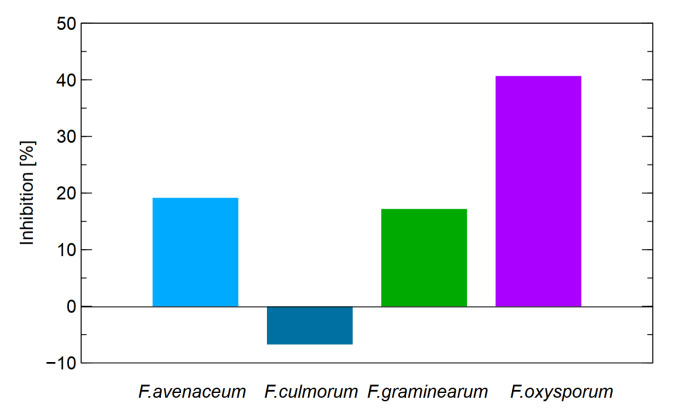
Inhibition of *Fusarium* growth via application of *Bacillus* bacteria treatment.

Research conducted by Stocka in her PhD thesis [23], supervised by Oszako, was focused on developing methods of biological control to combat air contamination with *Fusarium*, particularly in air conditioning and ventilation systems. It has been demonstrated that it is possible to use *Bacillus* bacteria, which are generally recognized as safe for humans, to inhibit the development of *Fusarium* mycelium. The research in question showed that the presence of bacteria significantly restricted the development of *F. avenaceum*, *F. graminearum*, *F. oxysporum*, and *F. poae*, but stimulated the growth of *F. culmorum*, so the effect, in this case, was the opposite to that which was desired (Figure A1 in the Appendix A). For this reason, it seems interesting to develop a low-cost method for identifying fungal species of the genus *Fusarium* using an electronic nose. The identification of species based on the volatile odors emitted could assist measures to reduce the occurrence of fungi not only in crops but also in building ventilation. The possible differentiation between various species of *Fusarium* may be important for the choice of preventive treatment.

We would like to draw attention here to some other research that deals with the detection or differentiation of *Fusarium* species via volatile organic compound analysis. Vikram et al. [24] used gas chromatography techniques. Falasconi et al. [25] investigated the detection of toxigenic strains of *F. verticillioides* in maize. Presicce et al. [26] reported on electronic nose studies of wheat contaminated by *F. poae* fungi. Perkowski et al. [27] used electronic nose analysis of VOC in wheat and triticale grains naturally infected and inoculated with *F. culmorum*. Eifler et al. [28] reported the differentiation between the species *F. graminearum* and *F. culmorum* in wheat grains using the SPME-GCMS method. Nordstorm et al. [29] utilized volatile organic compounds for early detection of *F. circinatum*. Feng et al. [30] used an electronic nose for early detection of *F. oxysporum* infection in tomato processing. Lebanska et al. [31] reported preliminary studies on the detection of *Fusarium* basal rot infections in onions and shallots. Camardo Leggieri et al. [32] reported on studies on the detection of *F. graminearum* mycotoxins in wheat.

There are several goals that we wanted to address in our research. First of all, we wanted to verify if the low-cost electronic nose can differentiate between several species of *Fusarium* fungi. This could have possible practical applications in the choice of preventive measures against contamination by these microorganisms, especially in buildings, air-conditioning, and ventilation equipment. Secondly, we wanted to verify some engineering aspects of the measurement technology: (i) What type of measurement method by the MOX sensors (i.e., gas composition modulation or sensor operation temperature modulation) is optimal for this application? (ii) What is the optimal time of data collection to obtain the best performance of the classification models based on the data obtained from the electronic nose measurements?

The manuscript is organized as follows: In Section 2.1 we describe the construction and functionality of the PW8 electronic nose device constructed in our laboratory. This is followed by Section 2.2, describing the measurements procedure, and Section 2.3, describing the signals collected during various modes of the sensor’s response. In Section 2.4, we describe fungi sample preparation. Then, in Section 2.5, we applied data analysis methods. The results of the measurements are presented in Section 3. In Section 3.1, we present results related to the possibility of differentiation between the studied species. Then, in Section 3.2 and Section 3.3, we present results related to the research questions concerning the choice of the sensor’s operation mode and data collection time, respectively. In Section 4.1, Section 4.2 and Section 4.3, we discuss these experimental results. A review of other research results concerning the analysis of chemical compositions of volatiles emitted by the *Fusarium* fungi is provided in Section 4.4. We summarize our findings in Section 5.

## 2. Materials and Methods

### 2.1. Electronic Nose Device

The electronic nose device used in our experiments was described in detail in the previous paper [33] and the papers describing its previous version, for which some parts are common [34,35,36]. For the reader’s convenience, we would like to include here short description of our electronic nose.

**Figure 1:**
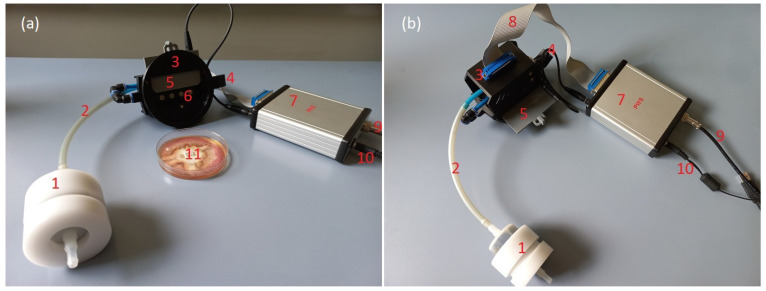
Measurement setup of the used electronic nose. (**a**) The device with a half-open sensor chamber shutter showing one row of sensors. (**b**) The device placed on a Petri dish with the sample. Charcoal filter (1) connected by pipe (2) to the sensor chamber (3), with the gas exhaust on the opposite side (4). The sensor chamber may be closed by a manually operated shutter (5). Sensors (6) may be exposed to the conditions of measured gas. Control unit (7) is connected to the sensors via signal cable (8), to the laptop via USB cable (9), and to a 12 V DC power supply (10). The measured sample is kept in a Petri dish (11).

In Figure 1, we present a photograph of the used device, demonstrating the area inside the sensor chamber and the device during the measurement process.

The constructed device consists of two main parts: first, the set of MOX sensors, which are placed in the sensor chamber, consisting of a probe which can be applied to the measured sample; second, the main electronic unit, which is connected to the computer.

Several metal oxide sensors of the TGS series from the Japanese company Figaro Co. are used in our device (Table A1 in the Appendix B). We selected all types of MOX sensors from this manufacturer which can work in two types of transient conditions: modulation of the gas composition and modulation of the sensor operation temperature.

The difference from our previous reports [33,36] is that we used only six sensors in the present experiment, as we found that only these could provide useful information on the nature of the fungal volatile organic compounds samples considered.

The gas sensor chamber [33] is equipped with a movable shutter, which allows it to open and expose the sensors to the measured volatiles. A simple pneumatic system is used to force a clean air flow inside the chamber to clean sensors between measurement cycles.

The ATmega 328P-PU microcontroller is used in the main unit of the device, which controls communication between the sensors and the computer.

The used MOX sensors operation requires in high-temperature conditions of several hundred degrees Celsius. Such a temperature is obtained by internal heaters which, according to the manufacturer’s specification [6], should be supplied by 5 Volts electrical power. The used sensors exhibit strong dependence on the heater voltage conditions [35]. The electrical circuit used has the functions of stabilizing the heater voltage at the required level [36] and modulating the heating voltage of the sensor, which enables modulation of the operating temperature of the sensors. These changes can be made in each individual sensor reading cycle.

A separate electric circuit [36] is used for measurement of the sensor’s response. Measurements with the electronic nose equipped with MOX sensors consist of reading the change in conductance of the sensor [6] during the transient response after changing the measurement conditions. The sensor conductance is measured via measurement of the voltage on a resistor serially connected to a sensor in the electrical circuit [37]. Since the meaning quantity is relative to the change of sensor conductance, one can use $U/U_{0}$ or $G/G_{0}$ interchangeably. The sensor reading cycles are repeated every 0.75 s. Two methods of generating transient conditions for the sensors are used for the measurements: (1) changing the gas composition in which the sensors are immersed, which is achieved by opening and closing the sensor chamber [33]; (2) changing the voltage of the sensor heater, which leads to a change in the operating temperature of the sensor [33,36].

**Figure 2:**
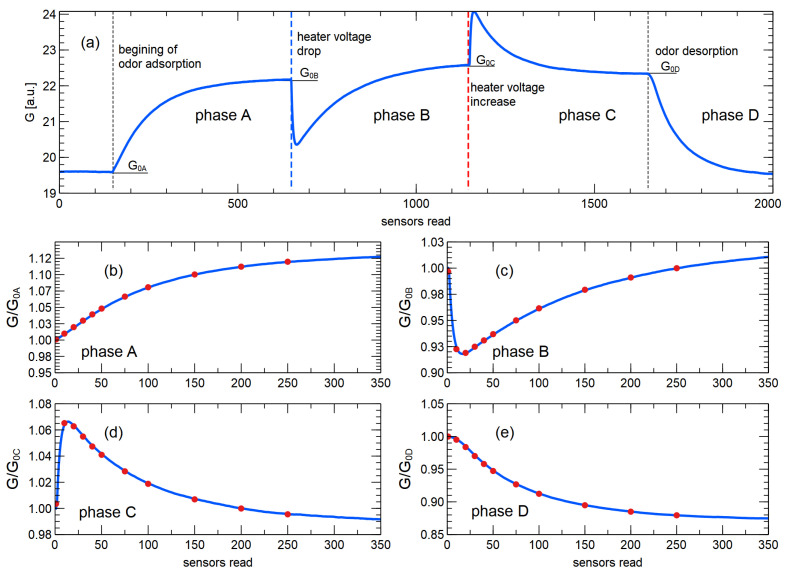
(**a**) Example of a sensor response curve during a measurement cycle. The four phases of data acquisition are indicated by the dashed rectangles and shown in the following sub-figures. (A) odor adsorption; (B) decrease in heating voltage; (C) increase in heating voltage; (D) odor desorption. Thee *x*-axis gives the sensor values (0.75 s). In sub-figures (**b**–**e**), the sensor values are counted from the beginning of the respective measurement phase. The *y*-axis in these subimages is shown as voltage normalized to the respective base value. The points in the subimages (**b**–**e**) represent time points at which modeling features were extracted.

A rectangular voltage drop/rise of 0.3 V with a duration of 500 sensor readings (6 min 15 s) was used for the sensor heating modulation mode (Figure 2a). The duration of the modulation was chosen to reach a steady state after the modulation step. The depth of the voltage modulation was chosen based on the experience of previous research [36].

### 2.2. Measurements with the Electronic Nose

#### 2.2.1. Measurement Cycle

The electronic nose, together with the Petri dishes overgrown with the fungus intended for the measurements, was kept in a laminar flow cabinet (Telstar, Bio II Advance) at 21 °C, with the air supply on all the time.

The measurements were carried out under the same conditions so that controlled temperature and humidity conditions could be maintained throughout the experiment.

At the beginning of the measurement, the chamber with the sensors (with the sensor chamber closed) was placed on an open Petri dish (Figure 1b). Each measurement cycle comprised 2100 sensor readings; each of the measurements lasted 0.75 s.

The measurement cycle started with forced clean air flow inside the gas chamber, which allowed us to determine the baseline conditions of the measurement cycle. The first 150 readings served as a reference point and allowed us to determine if the sensors were in a stationary state.

After that, the shutter of the chamber was opened manually and measurement continued. After 1500 measurements, the shutter of the chamber was closed, which started the forced clean airflow inside the chamber.

The last 450 measurement cycles were used to loosen and clean the sensors. The airflow inside the chamber was also maintained between the measurement cycles.

During the time, when the sensor chamber was opened, the heater voltage modulation occurred automatically (Figure 2), i.e., without the electronic nose operator intervention.

#### 2.2.2. Series of Measurements

Each day, a complete series of measurements was carried out for all samples. The order of the samples was selected at the beginning of each series using a random generator. When not measuring, all samples were covered in Petri dishes to avoid contamination.

Measurements were performed in two blocks of five days each. Each block included measurements on three dishes of each type, and odors from each dish were recorded only once per day. As these were measurements on living organisms, the readings changed every day due to the continuous growth of the fungus and the drying of the substrate on the dish. The first block covered the period from 10 to 14 July 2023, after which all samples were replaced with fresh ones and the next block of measurements began, which lasted from 17 to 21 July 2023.

The sensors were powered and heated throughout the experiment. We paid attention to their cleanliness. Thirty measurements were taken for each species. This gives a total number of 120 datasets to be analyzed.

### 2.3. Collecting Signals and Extracting Modeling Features

Figure 2a shows a schematic representation of the shape of the signal collected by the electronic nose sensor during the experiment. The magnitude of the signal represents the measured voltage, which corresponds to the conductance of the sensor. What is important and meaningful is not the exact value of this property, but its relation to the baseline $U/U_{0}$ [6]; so, in this figure, we can present this value in arbitrary units.

At the beginning of the measurement cycle, the electronic nose collects the measurements of senso conductance in the presence of clean air. In our case, this took about 2 min, and by observing the behavior of this part of the response, we could be sure that the response had a flat characteristic, which meant that the sensors were sufficiently cleaned and in a stable state.

As mentioned in Section 2.1, our electronic nose device can collect data in two types of sensor reactions: those caused by the change in gas composition and those caused by the change in the MOX sensor heating temperature. In Figure 2a, we marked four corresponding phases of the sensor response curve:

(A) When the sensor chamber is open and the sensors respond to the change in conditions from clean air to the presence of the odor;
(B) After reaching a steady state, the heating voltage decreases, which lowers the sensor temperature, and the response to this type of change is recorded;
(C) After reaching steady state, the heating voltage of the sensor is increased to the nominal voltage;
(D) When the sensor chamber is closed and the sensors respond to the change in gas composition from the presence of the measured odor to clean air conditions.

Since there are four measurement phases, we also used four different baseline values to normalize the sensor response, i.e., the quantity used for further analysis was the measured voltage divided by the voltage observed at the beginning of the phase: $U\left(t\right)/U_{0A-D}$ [33,36].

Such normalized responses were used to prepare classification predictors [38] for discriminating between the sample categories studied. Figure 2b–e shows the response of the sensor in these four measurement phases, normalized accordingly.

Three types of predictors were extracted from the sensor response curve.

- First, the magnitude of the normalized response at a selected time point;
- Second, the slope of the response curve at that time point (the slope was calculated using ±2 data points around the time point under consideration);
- Third, the area under the curve up to the selected time point.

The selected time points for predictor extraction were as follows: 1, 10, 20, 30, 40, 50, 75, 100, 150, 200, 250, and 300 readings (1 reading = 0.75 s), counted from the beginning of the corresponding phase of the measurement. It is worth noting that our electronic nose contained 6 sensors, which means that 18 predictors were extracted for a selected time point. We would also like to note that for the first time point considered, only the slope was extracted, as both the magnitude and the area under the curve do not contain useful information.

### 2.4. Samples Preparation

Four common fungal species of the genus *Fusarium* were selected for analyses: *F. avenaceum*, *F. culmorum*, *F. greaminarum*, and *F. oxysporum*. All fungal strains came from the collection of the Faculty of Agriculture and Forestry of the University of Warmia and Mazury in Olsztyn, Poland. The fungal samples were cultivated on a classical PDA agar medium (20 g dextrose, 15 g agar, 4 g potato starch, and 1 L distilled water) in 9 cm Petri dishes. Ten dishes were prepared for each species so that rapid replacement was possible in case of contamination or drying out of the substrate. The isolates were kept at room temperature until the mycelium had completely covered the surface of the dish until the measurement was carried out.

### 2.5. Classification Analysis

#### 2.5.1. Random Forest Classification Model

The main purpose of electronic nose measurements is to use the collected data to build classification models that are able to distinguish between categories of samples tested. Different classes of machine learning models can be used for data collected by sensors. In this work, the random forest (RF) [39] classification technique was used. This method has been successfully applied by other authors researching electronic noses [40,41,42,43,44] or analyzing data collected by different types of sensors.

Random forest is one of the most popular machine learning algorithms used for classification tasks. It belongs to the category of ensemble models. RF applies the creation of a large number of decision tree models, each of which is trained on a subset of the dataset as well as on a subset of predictors. The individual decision tree models are trained independently, and then the average of the results is used as the final output of the model RF. The ensemble estimator usually leads to significantly better performance of the joint model than each of the individual models. RF also leads to models that are less prone to overfitting.

The technique of RF offers several other important advantages. Since only subsets of the entire training dataset are used to train individual decision trees, the remaining data not used for training can be used as an independent observation set that can be used to estimate model performance. The so-called out-of-bag score (OOB) can be calculated for different measures to estimate the performance of the model. The OOB score is very similar in concept to the cross-validation method used to estimate the performance of statistical and machine learning models. It has been shown that OOB estimation converges with leave-one-out cross-validation [45].

#### 2.5.2. Models for Performance Measures

To evaluate model performance, we used three of the most common statistical measures, namely, accuracy, recall, and precision, which are defined in terms of the entries of the confusion matrix (Table 1).

Accuracy is defined as the proportion of correctly classified observations out of the total number of observations.

$$accuracy=\frac{tp+tn}{tp+tn+fp+fn}.$$

Recall is defined as the ratio of the number of correctly classified observations of a given category to the total number of observations in said category. It is focused on the possibility of detecting cases belonging to this category and is not penalized in cases wherein observations from other categories are incorrectly classified.

$$recall=\frac{tp}{tp+fn}.$$

Precision is defined as the ratio of the number of correctly classified observations in a given category to the number of observations classified as belonging to said category. This means that this measure is focused on the confidence that the classified observation truly belongs to the category in question.

$$precision=\frac{tp}{tp+fp}.$$

#### 2.5.3. Software Packages

The analysis of the data presented in this manuscript was carried out using computer codes developed in the Python 3.10 language. The scikit-learn package [46] was used for machine learning modeling.

## 3. Results

### 3.1. Recognition of Fusarium Species

**Figure 3:**
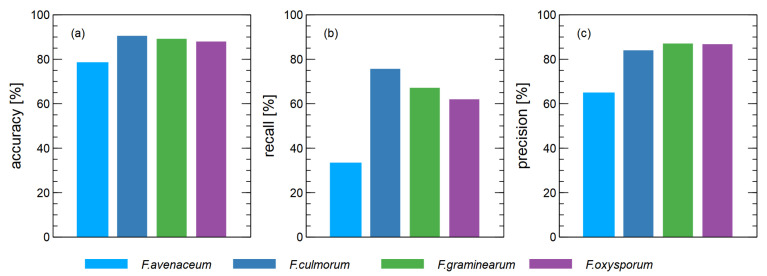
Performance of binary classification models to distinguish between *Fusarium* species (*x*-axis) and all other species. (**a**) accuracy, (**b**) recall, (**c**) precision.

In Figure 3, we show a comparison of the performance measures of four classification models for discriminating between one *Fusarium* species and all other species used in the experiment.

These results were obtained when all features extracted from the sensor data were used as predictors for the classification models. This means that the data were collected in all four different phases of the sensor response, as described in Section 2.3.

As can be seen, the difference between the cases considered is small when the accuracy of the classification is taken into account (Figure 3a), but it is noteworthy that the case of *F. avenaceum* is below the other cases. Moreover, the accuracy of *F. culmorum* as a classification target was better than the other species.

This performance of the recognition capabilities of one *Fusarium* species compared to another becomes even more evident when we consider other types of classification performance measures. In Figure 3b, where recognition is shown, we can see that the recognition of *F. avenaceum* is indeed the most problematic, as only 33% of the samples were recognized. For *F. culmorum*, on the other hand, the hit rate is 76%. For *F. greaminarum* and *F. oxysporum*, the hit rate is 67% and 62%, respectively, which is a relatively good result.

Looking at the precision of the binary classification models (Figure 3c), this performance measure gives results in the range of 84–87% for the three species, with good recognition performance, and 65% for the case of *F. avenaceum*.

### 3.2. Various Modes of Electronic Nose Response

**Figure 4:**
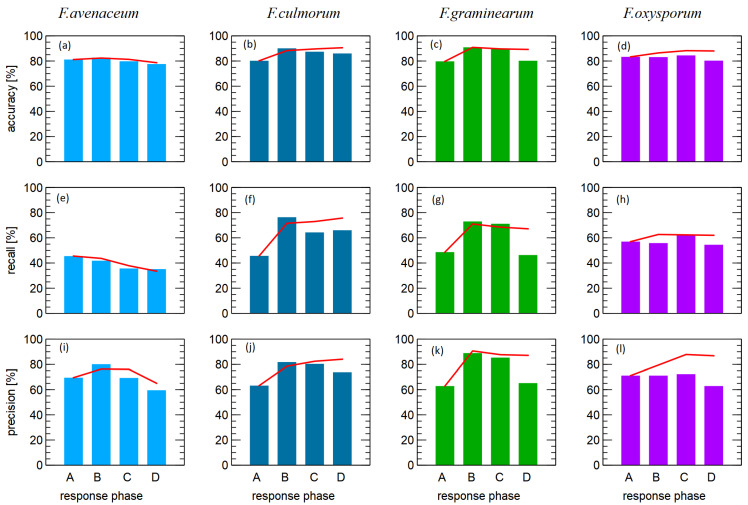
Performance of the binary classification models for one of the four *Fusarium* species considered compared to all others. Comparison of performance (bar graphs) when the predictors use data collected during one of the phases of the sensor response: (A) odor adsorption; (B) decrease in heating voltage; (C) increase in heating voltage; (D) odor desorption. The linear plots show the performance of the models when combinations of the predictors from multiple phases of the sensor response are used: A, A + B, A + B + C, and A + B + C + D.

A more detailed analysis of the results obtained is presented in Figure 4, where we compare the dependence of the classification performance on the different modes of electronic nose sensor response.

Let us now concentrate on the classification models with the aim of distinguishing *F. culmorum* from other *Fusarium* species. When examining the classification performance in Figure 4b,f,j, higher values were obtained when using the data collected in the reaction phase B of the sensors (voltage drop of the heater). This is true for the three considered performance measures of the model, but is most evident when considering recognition. The weakest classification performance for the three measures considered was obtained when using data collected in phase A (odor adsorption) of the sensor response.

When examining the results for discrimination between *F. greaminarum* and other species (Figure 4c,g,k), it was found that the best classification performance was obtained when data collected in the heating voltage change phases (B and C) were used as predictors.

When discriminating between *F. oxysporum* and other species (Figure 4d,h,l), the best performance was obtained for the sensor voltage drop (C), and it was most remarkable when we consider the recall measure.

Different patterns were observed when distinguishing between *F. avenaceum* and other species (Figure 4a,e,i) as in this case, the best classification performance was obtained for A phase (odor adsorption). However, since in the case of *F. avanaceum*, the overall recognition performance was much lower than that of the other species studied, we do not consider this result to be worthy of further investigation.

Figure 4 also shows the performance of the models for the cases where features obtained from more than one phase of the sensor response were used as model predictors. As can be seen here, the fusion of data from such different phases did not result in better model performance than that from data collected in the phases of the sensor response to the modulation of the heating voltage. The only exception was *F. oxysporum* and other species.

### 3.3. Electronic Nose Signals Collection Time

As explained in Section 2.3, the features used to build classification models were extracted from the raw response curve of the electronic nose sensors for several selected response time points. The total observation time in each phase of the sensor response was chosen so that the response reaches a steady state. The models presented in the previous sections were trained using features extracted from the data of up to 300 sensor readings, counting from the beginning of each considered phase of the sensor response.

**Figure 5:**
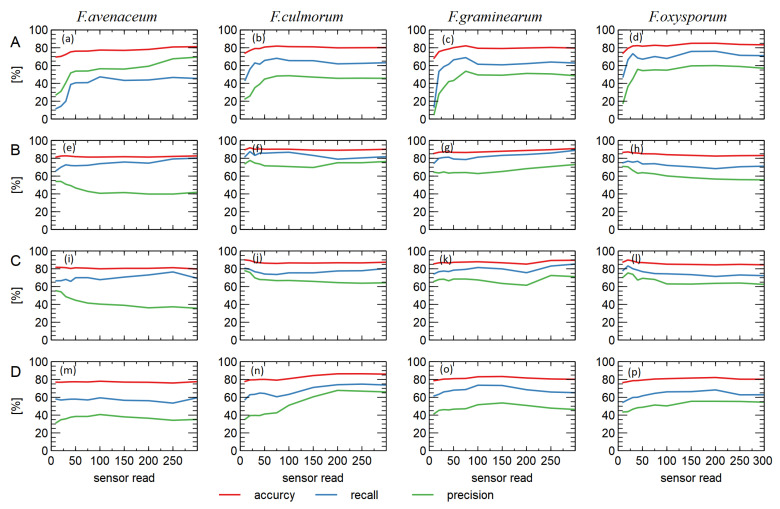
Performance of binary classification models for target discrimination between one of the *Fusarium* species and all others. Comparison of four phases of signal acquisition of the electronic nose sensor: (A) odor adsorption; (B) heating voltage drop; (C) heating voltage rise; (D) odor desorption. Comparison of the classification models for which features extracted from the data collected up to a certain moment of the sensor’s response were used as predictors (*x*-axis).

In our opinion, it was also interesting to check how the performance of the model depends on the time of data acquisition. To this task, we trained a set of classification models that applied different subsets of the full set of extracted predictors such that they were based on data collected up to a certain point in time. Figure 5 shows the individual results of such a set of classification models for each of the considered phases of the sensor response.

As can be seen here (Figure 5a–d), when looking at the response of the sensor to the change in composition of the gas in which the sensors were immersed (gas adsorption, phase (A)), the performance of the models increased and reached its optimum size after about 75 sensor readings (about 1 min). For the gas desorption phase (D), this time was longer; if we look at Figure 5n, it was about 200 sensor readings (2 min 30 s).

**Figure A2:**
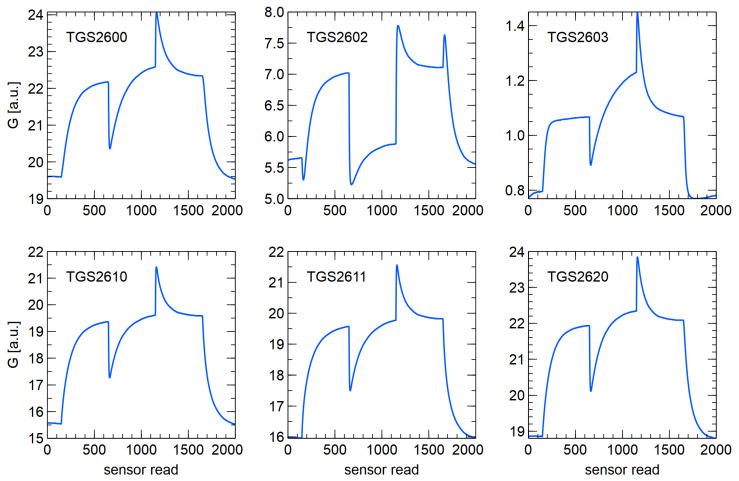
Example of collected electronic nose sensor’s responses during one cycle of a sample measurement. The sensor type is indicated inside the subfigures.

It is interesting to note that the response of the sensor in the mode of temperature modulation during data acquisition allowed for the training of classification models with optimal performance by using data acquired only at the beginning of the modulation phase, even in the case of models created with the data acquired during the first 3 s after the change in temperature of the sensor heating. These models were trained with six predictors, namely, the slopes of the sensor’s response curve, since other types of predictors are not used at this time (the magnitude of the normalized voltage is equal to one for all samples, and the area under the curve is equal to zero). An example of the responses of all sensors is shown in Figure A2 in Appendix C.

### 3.4. Linear Discrimination Analysis

**Figure 6:**
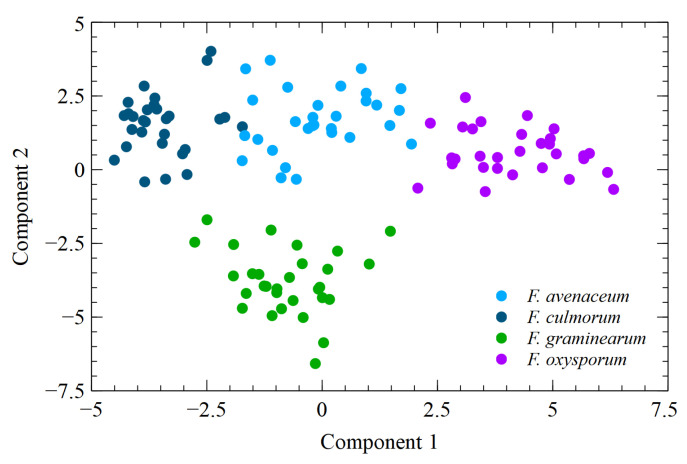
Dispersion of measurement samples in two components obtained as a projection of predictive features used for training classification models via linear discrimination analysis.

Figure 6 visualizes the dispersion of the points representing the measured samples. A two-dimensional projection of the predictors was used to build classification models.

As can be seen, an almost perfect differentiation between the sample categories could be achieved in the figure shown, and this is particularly clear in the case of *F. greaminarum*. At first sight, this seems to contradict the results presented above, where the reported classification accuracy was much lower, and the higher classification performance was obtained for *F. culmorum*. However, it should be borne in mind that Figure 6 was produced using all the data collected for the calculation of the dimensional projections. This means that such a perfect pattern of separation between different species was obtained for the case of overfitting. For the classification performance results presented in the above sections, we have given the results obtained using the out-of-bag random forests measurement, which incorporates the fact that the performance evaluation was conducted on data that were not used for model training.

The pattern visible in Figure 6 may nevertheless be useful in better understanding the results of the reported model classification performance. As can be seen here, the points representing *F. avanaceum*, even when clustered together, are located between clusters representing other species, namely, *F. culmorum* and *F. oxysporum*. This makes it impossible to achieve a linear separation between this species and all others. Even though random forest is not a linear separation model, this difficulty is reflected in the classification performance achieved.

## 4. Discussion

### 4.1. Differentiation between Fusarium Species via Electronic Nose Measurements

The results presented in Figure 3 demonstrate that by taking the signals collected by the electronic nose it was possible to create binary classification models allowing for the detection of *F. culmorum, F. greaminarum*, and *F. oxysporum* species with reasonable performance, although, for the case of *F. avenaceum*, the task is more difficult.

An interesting and encouraging result is that the highest detection performance, especially in terms of recall, was achieved for *F. culmorum*. Differentiation between this particular *Fusarium* species and others is especially important when we take into account the other results reported by Stocka [23]. *F. culmorum* interacts with *Bacillus* bacteria in the opposite manner to other *Fusarium* species: while the development of other species is inhibited by the presence of these bacteria, *F. culmorum* grows faster in their presence. This signifies that *Bacillus* bacteria cannot be used as a protective measure against this species of *Fusarium*, and detection in the case of that particular contamination agent has practical importance.

### 4.2. Choice of the Operating Mode of the Electronic Nose Sensor

In Figure 4, we compared the performance of classification models created using different types of response curves of the electronic nose sensor. An interesting pattern found is that the efficiency of discrimination between the *Fusarium* species considered was higher when we used data collected as a response of the sensor to the voltage change in the heater compared to the response in the adsorption and desorption phase.

One of the most important technical problems [7] in the design of electronic noses is the development of a precise and efficient method of changing the operating conditions of the sensor from pure air to the application of the measured gas. For this reason, efficient and precise modulation of the heating voltage is much easier to achieve with low-cost electronic noses.

The results obtained show that modulation of the heating voltage of the sensor during data acquisition allows for data acquisition that provides at least the same or better classification performance with respec tosamples of different *Fusarium* species.

### 4.3. Electronic Nose Data Collection Time

Examining the data presented in Figure 5, one might expect that the operating time of the electronic nose could be reduced. However, this issue is more complicated.

Looking at Figure 5a–d, which show the models created with data collected during the adsorption phase of the sensor reaction, one could deduce that the performance of the model did not improve after about 100 sensor readings (75 s). However, we believe that the duration of this phase of the sensor response should not be shortened, as we should wait until the sensors reach a steady state of response. As can be seen in Figure 2 (or in Figure A2 in Appendix C), this is reached after about 300 sensor readings (about 4 min). In our opinion, the steady state of the sensor response in the presence of the measured gas should be the state in which the heating modulation phase of the sensor is performed.

The results of the analysis presented in Figure 5e–l show that the main features used to build the classification models are extracted from the data collected at the beginning of the response of the sensor in the heating modulation mode. As can be seen from the data in Figure 2c,d, the time taken for the sensor to reach a steady state after the heating voltage is changed is also of the order of 300 sensor readings (about 4 min). However, if we decide to use only the data collected in phase B (decrease in the heating voltage) for modeling and disregard the data collected in phase C (increase in the heating voltage), we could reduce the data collection time during the operation of the electronic nose.

It should be noted here that we have not conducted experiments in which a phase of the sensor’s response curve (B, C, or D) started from conditions which were far from the sensor’s steady state. Therefore, we cannot assess what kind of information could be extracted from the sensor’s response when the heater’s voltage modulation starts from a transient condition. This is beyond the scope of our analysis and could be a subject of further investigation.

A reduction of the data acquisition time in phase D (gas desorption) could be achieved, as it can be seen in Figure 5m–p that the most important information useful for extracting features for the classification models is acquired at the very beginning of the sensor response. However, this does not allow us to reduce the operating time of the electronic nose. As can be seen in Figure 2e, cleaning the sensor after exposure to the measured gas also requires about 300 sensor readings (4 min) in order to reach a steady state in clear air.

Our results are in qualitative agreement with the conclusions of Rodriguez Gamboa et al. [47], who examined several publicly available datasets of electronic nose measurements and used machine models to demonstrate the potential of using only a portion of the electronic nose measurement data for correct odor classification. However, their analysis was based on data collected in the desorption/adsorption mode of the sensor.

### 4.4. Chemical Compounds Produced by Fusarium

*Fusarium* fungi cause plant diseases in a wide range of climatic conditions, occurring virtually all over the world. They infect trees and horticultural and agricultural plants, causing huge yield losses. Moreover, individual species are characterized by their unique abilities to produce mycotoxins from various groups, including trichothecenes, eniatins, fumonisins, and zearalenone [48], causing crop contamination and deterioration of its quality parameters.

Detection of *Fusarium* fungi both in the infectious phase on the plant and in agrifood processing products is possible via various diagnostic methods, including the classic breeding method, as well as immunological and genetic methods based on the PCR reaction or direct DNA sequencing [49,50]. The above-mentioned diagnostic methods are used with great success, but alternative methods are still being sought, the use of which could eliminate the disadvantages of currently used methods.

*Fusarium* fungi produce various chemical classes of VOCs, including alcohols, aldehydes, ketones, esters, and sesquiterpenes [51,52]. This diversity suggests the possibility of using unique VOC profiles characteristic of individual fungal species as a signature that will allow for the detection and identification of individual fungal species. Moreover, literature data indicate that it is possible to detect plant infections caused by *Fusarium*. As a result of the infection of maize plants with various *Fusarium* species (*F. graminearum, F. verticillioides*, and *F. subglutinans*), it was shown that the plants formed C6–C8 compounds and sesquiterpenoids [53,54]. VOCs were detected as early as 4–8 days after infection (dpi) until milk maturity. Additionally, the modification of the volatile profiles of maize plants after *Fusarium* infection was accompanied by the induction of plant defense compounds such as zealexins and oxylipins. These results reveal a broad metabolic response of the plant to pathogen attack. Volatile biomarkers of *Fusarium* infection have been indicated as promising indicators of plant infection by *Fusarium* in the asymptomatic phase.

The gold standard method used for analysis of the chemical composition of volatiles is gas chromatography coupled with the mass spectrometry method. However, the results of such a detailed analysis are not easily related to the chemical sensor’s response [55] and the electronic nose’s ability to detect and recognition of odors. The applied sensors are not specific and respond to various chemical components. The non-linear characteristic of the response makes it even more challenging when multiple volatiles are present in the emitted gases in complex compositions.

### 4.5. Future Applications

In the future, E-nose could become an important method for the correct diagnosis of the genus *Fusarium*. Individual species are characterized by a high degree of morphological similarity. In addition, distinguishing the species of *F. avenaceum, F. langsethiae, F. poae, F. sprotrichioides, and F. tricinctum* on the basis of microscopic features is a major challenge for researchers. Furthermore, in some cases, the molecular diagnostics of some *Fusarium* species using PCR techniques is also problematic. In such cases, gDNA sequencing should be used [56,57], which solves the problem of the low DNA polymorphism of marker genes between some species. Unfortunately, sequencing is a relatively expensive technique. In the case of e-nose validation against different species and strains of *Fusarium* with genetic traits, this technique can become an interesting alternative to molecular testing, which requires laboratory conditions, access to equipment, and personnel specialized in such tests. In addition, the development of the method may also concern the analysis of the toxinogenic capacity of fungi of the genus *Fusarium*. Individual strains of a species are often characterized by high variability, which means that the same *Fusarium* species can synthesize different mycotoxins [58]. The development of this interesting research direction could make E-nose an important tool for checking the mycotoxin contamination level of feed and food.

It should be noted here that the used MOX sensor’s response depends on environmental conditions such as temperature or humidity [6]. In our laboratory experiment, we paid attention to keeping these conditions constant using a flow chamber with the stabilization of these parameters. Construction of the device intended to be used outside of the laboratory would require the design of a stabilization system in the hardware. Another possible approach may be compensation [59] via some data analysis procedures.

## 5. Summary and Conclusions

An electronic nose with six non-specific gas sensors of the TGS series, manufactured by Figaro Inc. [6], was used to distinguish between four species of pathogenic *Fusarium* fungi: *F. avenaceum, F. culmorum, F. greaminarum*, and *F. oxysporum*.

One of the motivations for the research was the possibility of detecting fungal species whose growth could be inhibited by biological preventive measures through the application of *Bacillus* bacteria that are harmless to humans.

The transient response curve of the sensor was recorded at four different stages of the sensor response: (A) adsorption, i.e., when the gas content in which the sensors were immersed changed from pure air to sample gas conditions; (B) sensor heater voltage drop, i.e., when the sensors immersed in the sample gas changed their operating conditions by changing the temperature of the sensor element; (C) sensor heater voltage increase, i.e., when the temperature of the sensor was increased; (D) desorption mode, i.e., when the working conditions of the sensors changed from sample gas to pure air.

Random forest machine learning models were trained to classify the data, extracting features from the sensor’s response curves. Accuracy, precision, and detection were calculated as measurements of the model’s performance.

The best differentiation with binary classification models was achieved for the species *F. culmorum*. This result is very promising, as species recognition is of particular importance since the application of *Bacillus* does not reduce but in fact increases the growth of fungi.

It was shown that the signals collected with the sensor heating temperature modulation model allowed for the best classification performance. This is a very promising result as it simplifies and reduces the cost of the electronic nose. Precise and repeatable modulation of the sensor heating voltage is much easier to achieve than modulation of the gas composition. The former requires the use of a precise pneumatic system, which is not necessary in the case of the electronic nose, which mainly modulates the heating temperature.

As we observed when reviewing the available literature, most of the low-cost electronic noses are designed to utilize measurement methods with modulation of the gas composition. That is probably caused by the fact that most commercially available gas sensors are designed for such a mode of operation. In our research, we used the TGS series of sensors available via Figaro Inc offer [6], which, even if not designed and optimized for work in the modulation of the heater temperature mode, nonetheless allows us to achieve encouraging results in the application of electronic noses. It should also be noted that these sensors are produced with applications other than electronic noses in mind.

The possible optimization of the time taken during measurements of the electronic nose was investigated. We also investigated how long the measurements must take in order to achieve the best classification performance. It was found that the gas adsorption and desorption modes of the electronic nose require a longer time for data acquisition than the modulation modes of the heating voltage. It was found that in these modes, a very short time after changing the heating voltage was sufficient to collect data that contained most of the useful information for extracting features for sample classification. The last results indicate that the time for measuring the electronic nose can be significantly reduced, but this requires further research on the modulation of the heating voltage between the non-stationary states of the points of the response curve of the sensor.

## Figures and Tables

**Table 1:** The elements of the confusion matrix used to define the metric for the performance of the classification models.

		Predicted	
		**Positive**	**Negative**
actual	positive	tp (true positive)	fp (false negative)
	negative	fn (false positive)	tn (true negative)

## Data Availability

Not applicable.

## Appendix A. Inhibition of *Fusarium* Mycelium Development by the Presence of *Bacillus* Bacteria

In Figure A1, we present one of the results [23] demonstrating the influence of the *Bacillus* bacteria on the growth of various *Fusarium* species. The inhibition is defined as the percentage reduction in mycelium size growing on a Petri dish 48 h after application of the treatment. The results present the average values obtained when three species of *Bacillus* bacteria were used: *B. amyloliquefaciens*, *B. subtilis*, and *B. thuringiensis*. As one will notice, *F. culmorum* growth is intensified while, for other species, a reduction is observed. For all experiments with different species of *Bacillus* bacteria, the results were qualitatively similar, and for the purpose of this manuscript, the presentation of the average values is sufficient.

## Appendix B. TGS Sensors Used in the Electronic Nose Construction

**Table A1:** List of sensor models used in the electronic nose device and the target odors and gases [6].

Sensor	Target Gas Detection
TGS 2600	Highly sensitive to low concentrations of gaseous air contaminants such as hydrogen and carbon monoxide (for example, that in cigarette smoke). Can detect hydrogen at a level of several ppm.
TGS 2602	Highly sensitive to low concentrations of odorous gases such as ammonia and H${}_{2}$S generated from waste materials in office and home environments. Highly sensitive to low concentrations of VOCs such as toluene emitted from wood finishing and construction products.
TGS 2603	Highly sensitive to low concentrations of odorous gases such as amine-series and sulfurous odors generated from waste materials or spoiled foods such as fish.
TGS 2610	Uses filter material, eliminating the influence of interference gases such as alcohol. Highly selective of LP gas.
TGS 2611	Uses filter material, eliminating the influence of interference gases such as alcohol. Highly selective of methane gas.
TGS 2612	Highly sensitive to methane, propane, and butane. Targeted for LNG and LPG monitoring. Low sensitivity to alcohol vapors (a typical interference gas in the residential environment). The sensor is often used in consumer market gas alarms.
